# Effect of insurance status on mortality following surgical treatment of colorectal cancers in the United States

**DOI:** 10.1038/s41598-025-21334-6

**Published:** 2026-03-31

**Authors:** Atulya Aman Khosla, Aagamjit Singh, Muni Rubens, Venkataraghavan Ramamoorthy, Anshul Saxena, Sandeep Appunni, Krishna Raj Kunnath Rajappan, Tessa Ann Kanjiramkuzhey, Peter McGranaghan, Ishmael Jaiyesimi

**Affiliations:** 1 Department of Internal Medicine, Corewell Health William Beaumont University Hospital, Royal Oak, MI USA; 2 Department of Medical Oncology, Miami Cancer Institute, Baptist Health South Florida, Miami, FL USA; 3 Herbert Wertheim College of Medicine, Florida International University, Miami, FL USA; 4 Baptist Health South Florida, Miami, FL 33176 USA; 5 Government Medical College, Kozhikode, Kerala India; 6 Anthology Inc, Boca Raton, FL USA; 7 Duke University, Durham, NC USA; 8 Semmelweis Doctoral College, Semmelweis University, Budapest, Hungary; 9 Department of Hematology and Oncology, Corewell Health William Beaumont University Hospital, Royal Oak, MI USA; 10 Department of Cellular Therapy, Corewell Health William Beaumont University Hospital, 3601 W 13 Mile Rd, Royal Oak, MI 48073 USA

**Keywords:** Cancer, Oncology

## Abstract

Healthcare disparities in colorectal cancer, driven by insurance status and socioeconomic factors, lead to delayed diagnoses and poor surgical outcomes. We sought to examine the impact of insurance status on presentation, treatment, and in-hospital mortality among CRC patients undergoing colectomy using the Nationwide Inpatient Sample (NIS) database. We included patients aged 18–65 years diagnosed with colon cancer and undergoing colectomy, as identified by ICD-9-CM codes. We classified them as those with private insurance, Medicaid, or no insurance during the period January 1, 2005, through December 31, 2014. The primary independent variable was insurance status, and the primary outcome was in-hospital postoperative death. Associations between this outcome and insurance status were analyzed using the Cox proportional hazard model, both in the full cohort as well as in a subset of patients with low comorbidity, with models stratified by hospitals to account for clustering effects from variations in access to care. The study cohort included 301,304 patients, of whom 238,158 (79.0%) were privately insured, 40,417 (13.4%) on Medicaid, and 22,729 (7.6%) were uninsured. Most patients were White (71.6%), followed by African American (12.6%), Hispanic (8.4%), Asian/Pacific Islander (3.8%), and Native American (0.5%). A total of 55.4% of cases took place in teaching hospitals. In the unadjusted analysis, the mortality rate for privately insured patients was 0.7% (95% CI 0.6–0.7%) compared with 2.1% for uninsured patients (95% CI 1.7–2.5%) and 1.5% for Medicaid recipients (95% CI 1.2–1.8%; *p* = 0.001). After adjusting for patient characteristics and stratifying by hospital in patients with low comorbidity, uninsured patients still had a higher risk of experiencing in-hospital death (HR, 1.60; 95% CI 1.24–2.07) compared with privately insured patients, while no significant disparity was found in Medicaid recipients (HR, 0.95; 95% CI 0.75–1.22). Uninsured patients undergoing colectomy for colon cancer experienced the highest in-hospital mortality, a disparity not fully explained by overall health differences. These findings underscore the critical role of insurance coverage in improving surgical outcomes and highlight the need for policy interventions to reduce mortality disparities.

**Supplementary Information:**

The online version contains supplementary material available at 10.1038/s41598-025-21334-6.

## Introduction

The healthcare system in the USA, despite being one of the most expensive in the world, is also riddled with rising levels of inequality^1^. The gap in life expectancy between the richest 1% and poorest 1% of individuals rose between 2001 and 2014, at 14.6 years for men and 10.1 years for women^2^. Lack of health insurance is linked to reduced access to high-quality, affordable preventive and therapeutic cancer care^3^. Even insured cancer patients often experience significant financial hardship due to uncovered treatment costs, forcing trade-offs between medical care and basic needs, which can worsen health outcomes. The type of insurance plans can also affect the healthcare outcomes, especially in patients at the end of the bell curve in terms of disease severity and age^4^. It is important to bring these inequalities to light as a first step to mitigating them in the hope of equitable healthcare.

Outcomes in colorectal cancer, the third most common cancer in the US in terms of incident cases, highlight these stark inequalities^5^. Colorectal cancer screening facilitates early detection and intervention, reducing the risk of emergency presentations with advanced, complicated, or metastatic disease while increasing the likelihood of curative polypectomy or elective resection^6^. Non-elective surgeries are consistently associated with poorer outcomes, including prolonged hospitalization, higher complication rates, increased intensive care unit admissions, and greater mortality^6,7^. Despite overall advancements in colorectal cancer screening and treatment, substantial sociodemographic disparities persist^8^.

Prior studies on colorectal cancer outcomes in patients with and without insurance have shown significantly higher mortality rates in patients without insurance^4,9–11^. These differences may be attributed to differences in access to care and different treatment outcomes, possibly related to substandard care. Additionally, uninsured patients are more likely to present with advanced-stage disease, require emergency surgery, and experience lower overall survival compared to those with commercial insurance^12^. Similar patterns have been seen in breast cancer^13^ and brain tumors^14^.

To further bring to light the inequalities in cancer care, we decided to conduct a retrospective cohort study comparing outcomes following surgical (open or laparoscopic) treatment of colorectal cancer between those with private insurance, Medicaid, and the uninsured. We hypothesized that the uninsured and those with Medicaid would have worse outcomes despite undergoing similar surgeries. To test this, we analyzed data from the Nationwide Inpatient Sample (NIS), the largest all-payer inpatient database, and conducted a subset analysis of patients without major comorbidities to account for baseline health differences.

## Methods

### Study design and data source

In this retrospective observational study, data was gathered from the Nationwide Inpatient Sample (NIS) database and included the years 2005 to 2014. The NIS is a part of the Healthcare Cost and Utilization Project (HCUP) and is supported by a Federal-State-Industry partnership by the Agency for Healthcare Research and Quality (AHRQ). The NIS is the largest all-payer inpatient database in the United States. The NIS collects and stores data from nearly 40 million weighted hospitalizations annually. This includes a 20% stratified sample of discharge records from all U.S. hospitals, except acute-care hospitals, long-term care facilities, and rehabilitation centers.

### Study population

**Fig. 1:**
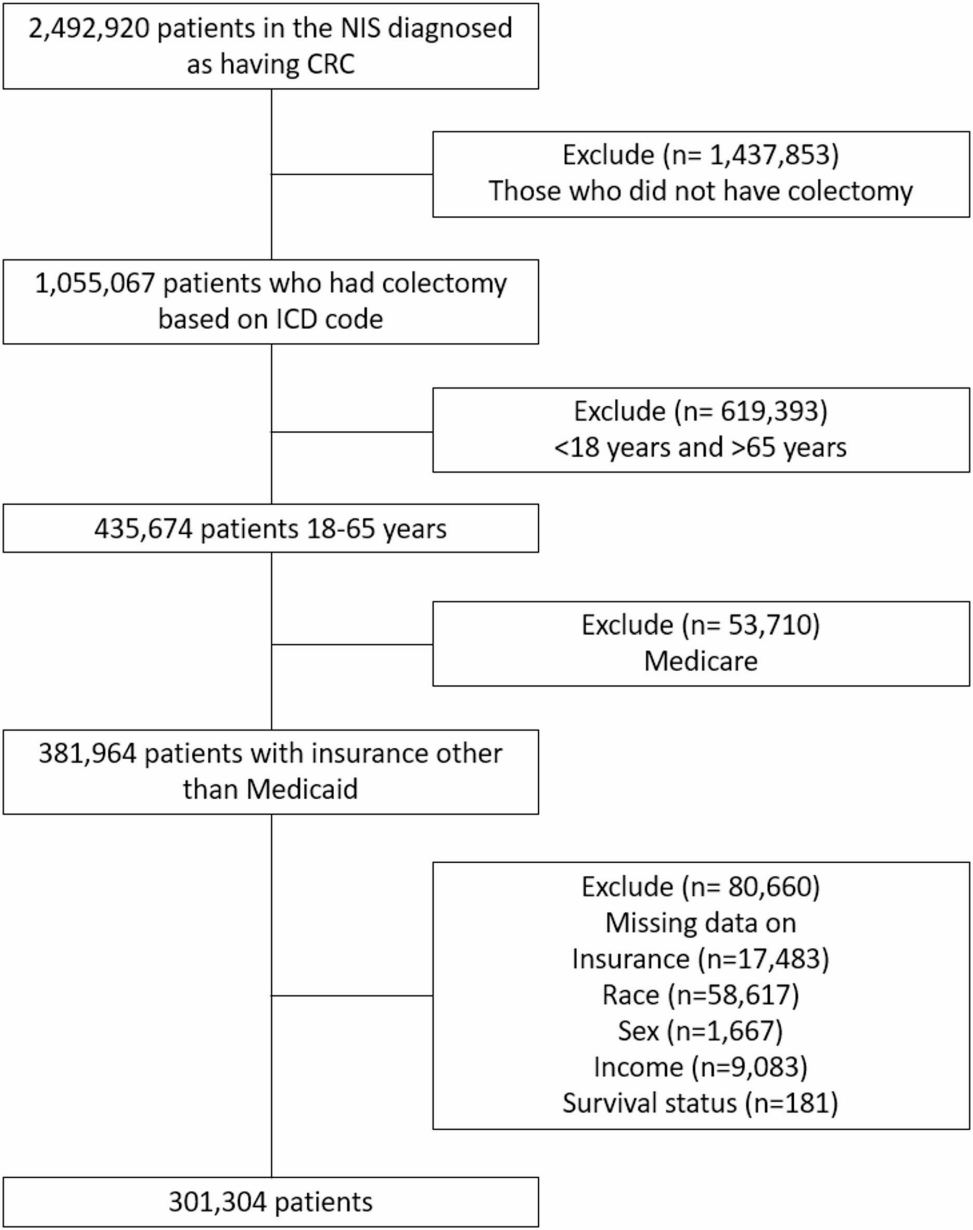
Study flowchart showing inclusion and exclusion criteria.

Adults 18 to 65 years of age who were hospitalized between 2005 and 2014 with a primary diagnosis of colon cancer and undergoing colectomy were identified from the NIS database using the International Classification of Diseases, Ninth Revision, Clinical Modification (ICD-9-CM) codes (Supplementary Table 1). The hospitalizations were classified based on their insurance status as those with private insurance, Medicaid, or no insurance. We did not have hospitalizations with Medicare because we included only individuals from 18 to 65 years, and patients younger than 65 years are generally ineligible for Medicare unless there are exceptional medical conditions such as amyotrophic lateral sclerosis, end-stage renal disease, or permanent disability for more than two years. In order to ensure rigorous critical appraisal and accurate interpretation of the findings, we adhered to the guidelines outlined in the Strengthening the Reporting of Observational Studies in Epidemiology (STROBE) recommendations^15^. Figure 1 shows the study flowchart.

### Study variables and outcome measures

The main outcome of this study was in-hospital postoperative death. This was defined as any death that occurred during the index hospitalization, regardless of length of stay. The event was captured from admission until discharge within the same hospitalization. Deaths that occurred after discharge, including during readmissions, were not included in this outcome definition. Demographic variables included in the study were hospitalization characteristics such as age, sex, race, and income. Other variables included cancer stage (not node positive/metastatic and node positive/metastatic) and surgery type (open and laparoscopic). Complications were classified as cardiovascular, gastrointestinal, infectious, mechanical, respiratory, surgical, urinary, and systemic. Hospital characteristics included hospital size, region, and location/teaching status. Comorbidity was assessed using the Elixhasuer comorbidity index. A low comorbidity index was defined as an Elixhasuer comorbidity index score of less than 3. Since this study involved only administrative data without any identifiable information, this study did not require informed consent.

### Statistical analysis

Descriptive statistics were used for showing demographic, hospital, and clinical characteristics stratified by insurance status and were reported as frequencies and percentages, initially in the full sample and subsequently in a subset with low comorbidity. Subsequently, unadjusted in-hospital mortality after surgery for colorectal cancer was assessed for the following variables: insurance status, age, sex, race, income, hospital region, hospital size, location/teaching status, cancer stage, surgery type, cardiovascular complications, gastrointestinal complications, infectious complications, wound complications, respiratory complications, surgical complications, urinary complications, and systemic complications. Cox proportional hazard models were used to identify factors associated with in-hospital postoperative death. Models were adjusted for demographic and socioeconomic characteristics, hospital characteristics, disease stage, surgery type, and complications following surgery. The time-to-event variable was defined as the number of days from the index admission date to the occurrence of in-hospital mortality (event) or discharge alive (censoring). Thus, the observation period was limited to the duration of the index hospitalization. We conducted a subgroup analysis among patients with low comorbidity admitted to urban teaching hospitals. The rationale for this subgroup analysis was to minimize potential confounding by severe comorbid conditions and institutional variation. Urban teaching hospitals were chosen because they typically provide specialized oncologic care and manage a large proportion of surgical cancer cases in the United States. Evaluating this relatively homogeneous subgroup allowed us to better isolate the effect of insurance status on mortality. The guidelines for using NIS data developed by Khera and Krumholz were used to ensure appropriate procedures for the study^16^. All tests were two-sided, and statistical significance was set at *p* < 0.05. SAS version 9.4 (SAS Institute, Cary, North Carolina) with complex survey data procedures was used for the analyses.

### Prior publication/presentation

An abstract of this manuscript was submitted for presentation to the ASCO Gastrointestinal Cancers Symposium 2025. Abstract published in Journal of Clinical Oncology and is available at https://ascopubs.org/doi/abs/10.1200/JCO.2025.43.4_suppl.40.

## Results

There was a total of 301,304 hospitalizations for colectomy for colon cancer. Among these hospitalizations, 79.0% had private insurance, 13.4% had Medicaid, and 7.6% did not have any insurance. The majority of hospitalizations (86.5%) were in the age group 45–65 years, and 53.9% were male. The majority of the hospitalizations were White (71.6%), followed by Black (12.6%), Hispanic (8.4%), and Asian (3.8%). Income was almost equally distributed among all four quartiles. The majority of the hospitalizations occurred in large (63.8%), urban teaching (55.4%) hospitals located in the South (40.4%). The majority were open surgery (82.4%), and 36.0% showed node positive/metastatic presentation. The most common complication was gastrointestinal (6.9%), followed by infection (4.4%), surgical (3.1%), and respiratory complications (2.0%). Table 1 shows the characteristics of the full study cohort by insurance status. The characteristics of the subset of patients with low comorbidity stratified by insurance status is shown in Table 2 and showed similar characteristics to the entire cohort except for a slightly higher private insurance (82.2%) and slightly lower Medicare (10.8%) and uninsured (6.9%) hospitalizations.

**Table 1:** Characteristics of the full study cohort, by insurance status.

Variables	Total (*N* = 301,304)	Private (*n* = 238,158)	Medicaid (*n* = 40,417)	Uninsured (*n* = 22,729)
	Weighted % (95% confidence interval)			
Insurance				
Private	79.0 (78.1–80.0)	–	–	–
Medicaid	13.4 (12.8–14.1)	–	–	–
Uninsured	7.6 (7.0-8.1)	–	–	–
Age				
18–44	13.5 (13.2–13.9)	12.7 (12.3–13.1)	17.8 (16.9–18.6)	14.8 (13.8–15.8)
45–65	86.5 (86.1–86.8)	87.3 (86.9–87.7)	82.2 (81.4–83.1)	85.2 (84.2–86.2)
Male	53.9 (53.5–54.4)	54.3 (53.8–54.7)	51.0 (49.9–52.1)	55.9 (54.4–57.4)
Race				
White	71.6 (70.6–72.6)	76.3 (75.4–77.2)	52.1 (50.0-54.2)	57.5 (54.7–60.3)
Black	12.6 (12.0-13.3)	10.4 (9.8–11.0)	22.3 (21.0-23.7)	18.9 (17.2–20.6)
Hispanic	8.4 (7.8–9.1)	6.5 (6.0-7.1)	15.5 (13.6–17.4)	15.7 (13.8–17.6)
Asian	3.8 (3.5–4.1)	3.4 (3.1–3.7)	5.8 (4.8–6.9)	3.6 (3.0-4.3)
Native	0.5 (0.4–0.6)	0.4 (0.3–0.5)	0.7 (0.5–0.9)	0.6 (0.3–0.8)
Other	3.1 (2.8–3.3)	2.9 (2.6–3.2)	3.5 (3.0–4.0)	3.7 (3.0-4.4)
Income				
Quart 1	23.8 (22.9–24.8)	19.7 (18.8–20.6)	40.1 (38.5–41.7)	37.8 (35.9–39.6)
Quart 2	24.2 (23.4–24.9)	23.3 (22.5–24.2)	27.3 (26.1–28.5)	27.4 (25.9–28.9)
Quart 3	25.0 (24.4–25.7)	26.2 (25.4–27.0)	20.1 (19.0-21.2)	21.5 (20.3–22.7)
Quart 4	27.0 (25.5–28.5)	30.7 (29.1–32.4)	12.5 (11.4–13.6)	13.4 (12.2–14.6)
Hospital region				
Northeast	21.5 (19.5–23.4)	22.0 (19.7–24.2)	23.6 (21.7–25.5)	12.5 (11.0-14.1)
Midwest	17.6 (16.4–18.8)	18.3 (17.0-19.7)	15.1 (13.8–16.4)	13.9 (11.3–16.5)
South	40.4 (38.4–42.4)	39.0 (36.8–41.3)	37.0 (34.8–39.3)	61.3 (58.2–64.4)
West	20.5 (19.1–22.0)	20.7 (19.0-22.3)	24.2 (21.8–26.7)	12.3 (10.8–13.7)
Hospital size				
Small	11.5 (10.6–12.4)	11.7 (10.7–12.7)	10.9 (9.9–11.9)	10.4 (9.2–11.5)
Medium	24.7 (23.3–26.0)	24.5 (23.0-25.9)	24.2 (22.5–26.0)	27.7 (25.1–30.3)
Large	63.8 (62.2–65.5)	63.8 (62.0-65.7)	64.9 (62.8–66.9)	62.0 (59.1–64.9)
Teaching status				
Rural	8.0 (7.4–8.6)	7.3 (6.7–7.8)	9.7 (8.9–10.5)	12.2 (11.1–13.3)
Urban non-teaching	36.6 (34.9–38.4)	37.9 (35.9–39.9)	30.6 (28.9–32.4)	34.1 (31.6–36.6)
Urban teaching	55.4 (53.5–57.3)	54.9 (52.7–57.0)	59.7 (57.6–61.8)	53.6 (50.5–56.8)
Node positive/metastatic	36.0 (35.4–36.6)	34.7 (34.1–35.4)	42.2 (40.8–43.5)	38.5 (37.1–40.0)
Surgery type				
Open	82.4 (81.8–83.1)	81.0 (80.3–81.7)	87.7 (86.9–88.5)	88.4 (87.3–89.4)
Laparoscopic	17.6 (16.9–18.2)	19.0 (18.3–19.7)	12.3 (11.5–13.1)	11.6 (10.6–12.7)
Complications				
Cardiovascular	1.5 (1.4–1.6)	1.4 (1.3–1.5)	2.0 (1.7–2.3)	1.5 (1.1–1.8)
Gastrointestinal	6.9 (6.6–7.3)	7.0 (6.6–7.4)	6.6 (6.0-7.2)	6.6 (5.8–7.4)
Infectious	4.4 (4.2–4.6)	4.0 (3.8–4.2)	6.3 (5.7–6.9)	4.9 (4.3–5.6)
Mechanical	1.0 (0.9–1.1)	1.0 (0.9–1.1)	1.4 (1.1–1.6)	0.7 (0.5–0.9)
Respiratory	2.0 (1.9–2.2)	1.9 (1.7-2.0)	2.9 (2.5–3.3)	2.1 (1.7–2.5)
Surgical	3.1 (2.9–3.2)	3.0 (2.9–3.2)	3.5 (3.1–3.9)	3.0 (2.5–3.5)
Urinary	1.0 (0.9–1.1)	1.0 (0.9–1.1)	1.0 (0.8–1.2)	1.0 (0.8–1.3)
Systemic	0.7 (0.6–0.8)	0.7 (0.6–0.8)	0.8 (0.6-1.0)	0.7 (0.4-1.0)
Comorbidities				
Hypertension	53.2 (52.5–53.9)	58.6 (58.1–59.0)	53.7 (53.0–54.5)	53.0 (52.3–53.7)
Diabetes mellitus	26.3 (25.8–26.8)	32.2 (31.9–32.5)	26.8 (26.3–27.3)	25.6 (25.1–26.1)
Anemia	35.3 (34.6–36.1)	26.4 (26.0–26.7)	36.5 (35.8–37.2)	36.2 (35.6–36.9)
Coagulation disorder	6.5 (6.2–6.8)	3.8 (3.7–3.9)	6.9 (6.6–7.2)	6.7 (6.4–7.0)
Pneumonia	18.8 (18.3–19.3)	12.5 (12.3–12.7)	19.4 (18.9–19.9)	18.9 (18.4–19.4)
Chronic renal disease	36.4 (35.7–37.2)	29.5 (29.0–30.1)	40.6 (39.9–41.3)	40.5 (39.8–41.2)
Dyslipidemia	25.1 (24.4–25.7)	33.1 (32.6–33.6)	27.1 (26.4–27.8)	26.0 (25.5–26.6)
Chronic lung disease	36.4 (35.8–36.9)	36.4 (36.0–36.7)	36.1 (35.5–36.7)	36.0 (35.5–36.6)
Liver disease	2.1 (2.0–2.2)	1.3 (1.3–1.4)	2.4 (2.2–2.5)	2.1 (2.0–2.3)
Fluid/electrolyte disorder	38.3 (37.7–38.9)	24.0 (23.7–24.3)	38.0 (37.3–38.6)	37.8 (37.2–38.4)
Tobacco use	11.5 (11.1–12.0)	14.6 (14.2–15.0)	12.4 (11.9–12.9)	12.1 (11.6–12.5)
Drug abuse	0.4 (0.3–0.4)	0.2 (0.2–0.3)	0.4 (0.3–0.5)	0.4 (0.3–0.5)
Alcohol abuse	53.2 (52.5–53.9)	58.6 (58.1–59.0)	53.7 (53.0–54.5)	53.0 (52.3–53.7)

Income variable provides a quartile classification of the estimated median household income of residents in the patient’s ZIP Code. The quartiles are identified by values of 1 to 4, indicating the poorest to wealthiest populations. These values are derived from ZIP Code-demographic data. Because these estimates are updated annually, the value ranges for this variable vary by year and adjusted for inflation.

Bedsize is classified as small, medium, or large within each U.S. region, and the cut-off points vary depending on the hospital’s location (Northeast, Midwest, South, West), teaching status (urban teaching, urban non-teaching, rural), and ownership/control. This classification is designed by the Agency for Healthcare Research and Quality to ensure comparability across different hospital types and regions.

**Table 2:** Characteristics of the subset of patients without comorbidity, stratified by insurance status.

Variables	Total (*N* = 212,550)	Private (*n* = 174,789)	Medicaid (*n* = 23,038)	Uninsured (*n* = 14,723)
	Weighted % (95% confidence interval)			
Insurance				
Private	82.2 (81.2–83.3)	–	–	–
Medicaid	10.8 (10.1–11.5)	–	–	–
Uninsured	6.9 (6.3–7.5)	–	–	–
Age				
18–44	15.4 (15.0-15.8)	14.4 (13.9–14.8)	22.3 (21.1–23.6)	17.2 (15.9–18.6)
45–65	84.6 (84.2–85.0)	85.6 (85.2–86.1)	77.7 (76.4–78.9)	82.8 (81.4–84.1)
Male	54.8 (54.3–55.3)	55.0 (54.4–55.5)	52.3 (50.9–53.8)	56.5 (54.7–58.3)
Race				
White	73.3 (72.2–74.4)	77.4 (76.5–78.3)	52.1 (49.3–55.0)	58.0 (54.7–61.4)
Black	10.6 (10.1–11.2)	9.0 (8.5–9.6)	19.1 (17.6–20.6)	16.5 (14.4–18.7)
Hispanic	8.2 (7.5-9.0)	6.4 (5.8-7.0)	17.0 (14.4–19.7)	16.5 (14.2–18.8)
Asian	4.2 (3.8–4.6)	3.8 (3.4–4.1)	7.2 (5.8–8.7)	4.4 (3.5–5.3)
Native	0.5 (0.3–0.6)	0.4 (0.3–0.5)	0.6 (0.4–0.9)	0.6 (0.3–0.9)
Other	3.1 (2.8–3.5)	3.0 (2.6–3.3)	3.8 (3.2–4.5)	3.9 (3.1–4.8)
Income				
Quart 1	21.8 (20.8–22.8)	18.4 (17.5–19.3)	37.9 (35.8–39.9)	36.4 (34.2–38.5)
Quart 2	23.8 (22.9–24.6)	22.9 (22.0-23.8)	27.7 (26.1–29.2)	27.4 (25.6–29.1)
Quart 3	25.4 (24.7–26.2)	26.3 (25.5–27.1)	21.0 (19.7–22.3)	22.1 (20.6–23.7)
Quart 4	29.1 (27.4–30.7)	32.4 (30.6–34.2)	13.4 (12.1–14.8)	14.1 (12.6–15.6)
Hospital region				
Northeast	22.8 (20.5–25.0)	23.1 (20.6–25.6)	26.0 (23.5–28.5)	13.6 (11.9–15.4)
Midwest	17.0 (15.7–18.3)	17.8 (16.3–19.2)	13.4 (11.9–14.8)	14.1 (10.6–17.5)
South	39.3 (37.0-41.5)	38.1 (35.7–40.5)	34.8 (31.9–37.6)	59.8 (56.1–63.5)
West	20.9 (19.3–22.6)	21.0 (19.2–22.8)	25.9 (22.3–29.5)	12.5 (10.8–14.2)
Hospital size				
Small	11.4 (10.5–12.4)	11.6 (10.5–12.6)	10.9 (9.6–12.3)	10.5 (9.1–11.8)
Medium	24.2 (22.8–25.7)	24.1 (22.5–25.6)	23.5 (21.2–25.7)	27.2 (24.2–30.2)
Large	64.4 (62.5–66.2)	64.4 (62.4–66.4)	65.6 (62.9–68.3)	62.3 (58.9–65.7)
Teaching status				
Rural	7.9 (7.2–8.5)	7.2 (6.6–7.8)	9.7 (8.7–10.6)	13.1 (11.8–14.5)
Urban non-teaching	35.7 (33.8–37.6)	37.0 (34.9–39.2)	28.0 (25.9–30.0)	32.1 (29.3–34.9)
Urban teaching	56.4 (54.3–58.5)	55.8 (53.5–58.1)	62.3 (59.8–64.9)	54.7 (51.1–58.3)
Node positive/metastatic	30.1 (29.4–30.8)	29.4 (28.7–30.2)	34.1 (32.5–35.8)	32.0 (30.2–33.8)
Surgery type				
Open	81.7 (81.0-82.4)	81.7 (81.0-82.4)	87.5 (86.4–88.6)	87.5 (86.4–88.6)
Laparoscopic	18.3 (17.6–19.0)	18.3 (17.6–19.0)	12.5 (11.4–13.6)	12.5 (11.4–13.6)
Complications				
Cardiovascular	1.0 (0.9–1.1)	1.0 (0.9–1.1)	1.6 (1.2–1.9)	1.0 (0.7–1.4)
Gastrointestinal	6.5 (6.1–6.9)	6.5 (6.1–6.9)	6.3 (5.5-7.0)	6.6 (5.7–7.6)
Infectious	3.4 (3.3–3.6)	3.1 (2.9–3.3)	5.4 (4.8–6.1)	4.2 (3.4-5.0)
Mechanical	0.8 (0.7–0.9)	0.8 (0.7–0.9)	1.0 (0.7–1.3)	0.7 (0.4–0.9)
Respiratory	1.3 (1.2–1.5)	1.3 (1.1–1.4)	1.9 (1.5–2.3)	1.3 (0.9–1.6)
Surgical	2.8 (2.7-3.0)	2.8 (2.6-3.0)	3.2 (2.7–3.7)	2.5 (1.9–3.2)
Urinary	0.9 (0.8-1.0)	0.9 (0.8-1.0)	1.1 (0.8–1.3)	0.9 (0.6–1.3)
Systemic	0.6 (0.6–0.7)	0.6 (0.5–0.7)	0.8 (0.5-1.0)	0.5 (0.3–0.7)

Unadjusted analysis among the full sample showed that after surgery for colorectal cancer, in-hospital mortality rates were 2.1% (1.7–2.5%) among the uninsured, 1.5% (1.2–1.8%) among those on Medicaid, and 0.7% (0.6–0.7%) among those with private insurance. In the cohort with low comorbidity, in-hospital mortality rates were 1.1% (0.7–1.5%) among the uninsured, 0.9% (0.6–1.1%) among those on Medicaid, and 0.3% (0.3–0.4%) among those with private insurance. Results from the full sample, as well as those with low comorbidity, showed that in-hospital mortality rates were significantly higher among uninsured and Medicaid recipients compared to those with private insurance (Table 3).

**Table 3:** Unadjusted in-hospital mortality after surgery for colorectal cancer.

Characteristic	Full Sample (*N* = 301,304)		Cohort With Low Comorbidity	
	% (95% CI)	*P* value	% (95% CI)	*P* value
Insurance		< 0.001		< 0.001
Uninsured	2.1 (1.7–2.5)		1.1 (0.7–1.5)	
Medicaid	1.5 (1.2–1.8)		0.9 (0.6–1.1)	
Private	0.7 (0.6–0.7)		0.3 (0.3–0.4)	
Age		< 0.001		0.017
18–44	0.4 (0.2–0.5)		0.3 (0.1–0.4)	
45–65	1.0 (0.9-1.0)		0.5 (0.4–0.5)	
Sex		< 0.001		0.559
Male	1.0 (0.9–1.1)		0.4 (0.4–0.5)	
Female	0.7 (0.6–0.8)		0.4 (0.3–0.5)	
Race		< 0.001		0.040
White	0.8 (0.7–0.9)		0.4 (0.4–0.5)	
Black	1.3 (1.0-1.6)		0.7 (0.4–0.9)	
Hispanic	0.9 (0.6–1.1)		0.2 (0.1–0.4)	
Asian	0.8 (0.4–1.1)		0.2 (0.0-0.4)	
Native	0.3 (0.0-0.9)		0.5 (0.0-1.4)	
Other	0.5 (0.2–0.8)		0.3 (0.0-0.6)	
Income		< 0.001		< 0.001
Quart 1	1.2 (1.0-1.4)		0.7 (0.5–0.8)	
Quart 2	0.9 (0.8–1.1)		0.5 (0.4–0.7)	
Quart 3	0.7 (0.6–0.9)		0.3 (0.2–0.4)	
Quart 4	0.7 (0.5–0.8)		0.3 (0.2–0.4)	
Hospital region		0.009		0.199
Northeast	0.7 (0.6–0.9)		0.4 (0.3–0.6)	
Midwest	0.8 (0.6–0.9)		0.3 (0.1–0.4)	
South	1.0 (0.9–1.2)		0.5 (0.4–0.6)	
West	0.8 (0.7-1.0)		0.4 (0.3–0.6)	
Hospital size		0.809		0.294
Small	0.8 (0.6–1.1)		0.5 (0.3–0.7)	
Medium	0.8 (0.7-1.0)		0.5 (0.4–0.6)	
Large	0.9 (0.8-1.0)		0.4 (0.3–0.5)	
Location/teaching status		0.001		0.006
Rural	1.3 (1.0-1.7)		0.8 (0.4–1.2)	
Urban non-teaching	0.9 (0.8-1.0)		0.4 (0.3–0.5)	
Urban teaching	0.8 (0.7–0.9)		0.4 (0.3–0.5)	
Stage		< 0.001		< 0.001
Not node positive/metastatic	0.6 (0.5–0.6)		0.3 (0.2–0.4)	
Node positive/metastatic	1.4 (1.2–1.6)		0.7 (0.6–0.9)	
Surgery type		< 0.001		< 0.001
Open	1.0 (0.9–1.1)		0.5 (0.4–0.6)	
Laparoscopic	0.2 (0.1–0.2)		0.0 (0.0-0.1)	
Cardiovascular complications		< 0.001		< 0.001
No	0.8 (0.7–0.9)		0.4 (0.3–0.4)	
Yes	6.4 (4.8-8.0)		4.7 (2.9–6.5)	
Gastrointestinal complications		< 0.001		< 0.001
No	0.8 (0.8–0.9)		0.4 (0.3–0.5)	
Yes	1.3 (1.0-1.7)		0.9 (0.5–1.2)	
Infectious complications		< 0.001		< 0.001
No	0.8 (0.7–0.9)		0.4 (0.3–0.4)	
Yes	2.4 (1.8-3.0)		1.7 (1.0-2.3)	
Wound complications		< 0.001		< 0.001
No	0.9 (0.8–0.9)		0.4 (0.4–0.5)	
Yes	2.1 (1.1–3.1)		1.6 (0.4–2.7)	
Respiratory complications		< 0.001		< 0.001
No	0.8 (0.7–0.8)		0.4 (0.3–0.4)	
Yes	5.9 (4.5–7.3)		5.1 (3.2–6.9)	
Surgical complications		< 0.001		< 0.001
No	0.8 (0.8–0.9)		0.4 (0.3–0.5)	
Yes	2.1 (1.5–2.8)		1.2 (0.6–1.8)	
Urinary complications		0.040		0.008
No	0.9 (0.8–0.9)		0.4 (0.4–0.5)	
Yes	1.6 (0.7–2.6)		1.3 (0.2–2.4)	
Systemic complications		< 0.001		< 0.001
No	0.8 (0.8–0.9)		0.4 (0.3–0.5)	
Yes	6.3 (4.2–8.5)		4.3 (1.8–6.8)	

Among the full sample, all demographic, hospital, and clinical characteristics were significantly associated with in-hospital mortality except for hospital size (*p* = 0.809). Likewise, among the subset with low comorbidity, all demographic, hospital, and clinical characteristics were significantly associated with in-hospital mortality except for hospital size (*p* = 0.294), sex (*p* = 0.559), and hospital region (*p* = 0.199).

Cox proportional regression analysis showed that risk of in-hospital mortality after surgeries for colorectal cancer was significantly higher among uninsured, compared to private insurance in the full sample (HR, 1.60; CI 1.24–2.07), as well as among the cohort with low comorbidity (HR, 2.12; CI 1.42–3.15), and the cohort with low comorbidity and admitted to urban teaching hospitals (HR, 2.28; CI 1.25–4.16). However, the risk of in-hospital mortality did not differ between Medicaid and private insurance among any of the cohorts (Table 4). Please see Supplementary Table 2 shows the complete model for full sample, Supplementary Table 3 shows the complete model for low comorbidity, and Supplementary Table 4 shows the complete model for low comorbidity in urban teaching hospitals.

**Table 4:** In-hospital mortality after surgery for a colorectal cancer.

Covariate	Full sample	Cohort with low comorbidity	Cohort with low comorbidity in urban teaching hospitals
	Hazard ratio (95% confidence interval)		
Insurance			
Private	Reference	Reference	Reference
Medicaid	0.95 (0.75–1.22)	1.38 (0.91–2.11)	1.66 (0.91–3.02)
Uninsured	1.60 (1.24–2.07)	2.12 (1.42–3.15)	2.28 (1.25–4.16)

Hazard Ratio (95% confidence interval) are from cox proportional regression using sampling and poststratification weights and hospital clusters, yielding nationally representative estimates for the U.S. population. The models included demographic and socioeconomic characteristics, hospital characteristics, disease stage, surgery type, and complications following surgery. For complete model see Supplementary Table 2, Supplementary Tables 3, and Supplementary Table 4.

## Discussion

Our study shows that the in-hospital mortality rate for post-colorectal surgery patients from the NIS database is significantly higher in those who are uninsured compared to those with private insurance of any type (HR 1.60, 95% CI 1.24–2.07). The difference was not significant for Medicaid compared to private insurance (HR 0.95, 95% CI 0.75–1.22).

There can be various reasons why the mortality rates are higher in patients without health insurance. This could be due to uninsured patients receiving the suboptimal quality of care, or having limited access to care, or delayed care^14,17^. We tried to adjust for the first by comparing outcomes in patients with low comorbidity. Prior studies have shown that patients without health insurance receive substandard care compared to their insured counterparts^18,19^. There was no way to directly assess the quality of care received by patients from the NIS database.

A retrospective study using the NSQIP database revealed that among patients with CRC undergoing surgery at a safety net hospital, the Medicaid/uninsured patients had higher odds of presenting with pre-operative serious acute conditions (aOR 2.02, 95% CI 1.22–3.52, *P* = 0.009) and undergoing urgent or emergent operations (aOR 1.80, 95% CI 1.28–2.55, *P* < 0.001) than privately insured patients^20^. A recent study using the NCDB database revealed that in patients with gastrointestinal malignancies, those with private insurance were more likely than Medicare patients to have negative margins (OR 1.08, 95% CI 1.06–1.10) and adequate lymphadenectomy (OR 1.06, 95% CI 1.04–1.06), whereas uninsured patients had the lowest likelihood of achieving negative margins (OR 0.78, 95% CI 0.75–0.81) and adequate lymphadenectomy (OR 0.95, 95% CI 0.92–0.99)^11^. A NIS database analysis for patients with colon cancer undergoing minimally invasive surgery revealed lower odds of undergoing the procedure (OR 0.85, 95% CI 0.74–0.97, *p* = 0.017) and higher odds of postoperative genitourinary complications (OR 1.36, 95% CI 1.08–1.71, *p* = 0.009) among Medicaid patients^4^. Moreover, Medicaid coverage was associated with a 1.76-day longer LOS (*p* < 0.001) and $5,043 higher hospitalization costs (*p* < 0.001)^4^. In another recent study using NIS data, Allar et al. demonstrated that lack of insurance and residence in a low-income ZIP code were associated with significantly increased odds of non-elective surgery (OR 4.54, 95% CI 4.11–5.02) and inpatient blood transfusions (OR 1.51, 95% CI 1.34–1.70) compared to patients with commercial insurance residing in high-income ZIP codes^21^. Among these factors, insurance status exerted a greater influence on surgical urgency and transfusion rates^21^. Although it is essential to examine the independent effects of insurance status and socioeconomic status on clinical outcomes, these variables frequently interact, as individuals in lower-income areas are disproportionately uninsured, compounding their impact on healthcare access and surgical risk.

While we only looked at patients who underwent surgery for colorectal cancer, in a study including all incident cases of colorectal cancer in Florida, the relative risk of death for the uninsured compared to those with commercial fee-for-service insurance was 1.4 (95% CI, 1.12, 1.77), and for those with Medicaid compared to the same was 1.44 (95% CI,1.06, 1.97)^9^. This effect was partly explained by a lower chance of receiving definitive surgery in the uninsured and Medicaid groups, thus indicating worse access to care in these groups. Although we adjusted for disease stage, surgery type, and complications following surgery, our results may be partly explained by delayed care for the uninsured.

Our study had a sample size of over 300,000 patients and was adequately powered to obtain statistically meaningful results. The NIS database offers the possibility to avoid statistical clustering by stratifying at the hospital level, which we used in our study. We also adjusted for other variables that can independently affect mortality, like co-morbidities, socio-economic status, hospital characteristics, disease stage, surgery type, and complications following surgery. This study has several important limitations. First, it is difficult to assess for true co-morbidities in patients without insurance due to less available information in the medical records. This may lead to confounding, as patients labeled “low comorbidity” may actually have unknown co-morbidities, which may independently increase mortality. Second, we only evaluated in-hospital mortality, while deaths occurring soon after discharge also reflect the quality of post-operative care and should be considered for a fair analysis. Third, some uninsured patients enroll in Medicaid during the hospital stay, and they are classified as Medicaid in the NIS database, although they would be more representative of the uninsured group. Lastly, we could not assess the time from diagnosis to surgery or data on cancer stage, which has an important bearing on mortality and limits the ability to fully characterize disease severity at presentation.

Our study shows a significantly higher mortality rate for uninsured patients compared to patients with private insurance after surgery for colorectal cancer and a more pronounced effect in urban teaching hospitals. These effects persisted after adjusting for demographic and socioeconomic characteristics, hospital characteristics, disease stage, surgery type, and complications following surgery. Financial and racial disparities in healthcare remain a major concern that needs to be addressed and managed to uphold the basic human right to health. Having no insurance certainly increases mortality in colorectal cancer, as seen in our study, but also in patients with breast cancer, post-lung resection, post gastrointestinal procedures, and post CABG. Thus, efforts should be made to improve health insurance coverage in the country. The Affordable Care Act helped improve access to health care by expanding insurance coverage to around 25 million previously uninsured Americans. The remaining uninsured 25 million people in the country are more likely to forego medical care compared to their insured counterparts, and this may lead to overall increased healthcare costs down the line in the form of higher out-of-pocket payments. There is a need for more studies highlighting the inequalities in healthcare and healthcare reforms to mitigate the same.

Our study is limited because we used data from 2004 to 2015 which predates the widespread adoption of minimally invasive and robotic surgical techniques as well as Enhanced Recovery After Surgery protocols. While these advances have improved perioperative outcomes, our primary focus was to evaluate disparities in in-hospital mortality by insurance status.

## Conclusions

Our study demonstrates that uninsured patients undergoing colorectal cancer surgery experience significantly higher in-hospital mortality rates compared to those with private insurance, with this disparity being more pronounced in urban teaching hospitals. These findings persist even after adjusting for demographic, socioeconomic, and clinical factors, underscoring the impact of financial barriers on surgical outcomes. While our analysis accounts for key confounders, limitations such as unmeasured comorbidities and the inability to assess preoperative delays highlight the need for further research to elucidate the mechanisms driving these disparities. Beyond colorectal cancer, the adverse effects of a lack of insurance have been documented across multiple malignancies and surgical procedures, reinforcing the broader implications of healthcare inequity. Expanding insurance coverage has the potential to reduce disparities in surgical outcomes and improve overall healthcare access. While policies like the Affordable Care Act have extended coverage to millions, a substantial uninsured population remains at risk. Future efforts should focus on healthcare reforms aimed at closing these gaps, improving early access to care, and ensuring equitable surgical outcomes.

## Supplementary Information

Below is the link to the electronic supplementary material.

Supplementary Material 1.

## Data Availability

Data is publicly available for purchase at: https://hcup-us.ahrq.gov/db/nation/nis/nisdbdocumentation.jsp . Please contact corresponding author at mrube001@fiu.edu for further details.
