# Supplementary material for: Effect of insurance status on mortality following surgical treatment of colorectal cancers in the United States
Source: Sci Rep. 2026 Mar 31;16:10643. doi: 10.1038/s41598-025-21334-6 (PMC13039124; doi:10.1038/s41598-025-21334-6)
Supplement: Supplementary file 1 — Supplementary Material 1. [file 41598_2025_21334_MOESM1_ESM.docx]

Supplementary Table 1. ICD-9-CM diagnosis and procedure codes

| **Condition** | **ICD-9-CM Diagnosis and Procedure Codes** |
| --- | --- |
| Colon cancer | 1530, 1531, 1532, 1533, 1534, 1535, 1536, 1537, 1538, 1539, 1540, 1541, and 1548 |
| Open surgery | 4571, 4572, 4573, 4574, 4575, 4576, 4577, 4578, 4579, 4582, 4583, 4840, 4841, 4842, 4843, 4844, 4845, 4846, 4847, 4848, 4849, 4850, 4851, 4852, 4853, 4854, 4855, 4856, 4857, 4858, 4859, 4861, 4862, 4863, 4864, 4865, 4866, 4867, 4868, and 4869 |
| Laparoscopic surgery | 1731, 1732, 1733, 1734, 1735, 1736, 1737, 1738, 1739, 4581, 4842, and 4851 |
| Node positive and metastatic disease | 196, 1960, 1961, 1962, 1963, 1964, 1965, 1966, 1967, 1968, 1969, 197, 1970, 1971, 1972, 1973, 1974, 1975, 1976, 1977, 1978, 198, 1980, 1981, 1982, 1983, 1984, 1985, 1986, 1987, 1988, 19881, 19882, 19889, and 1991 |
|  |  |
| Cardiovascular complications | 99779, 41511, 99702, 9972, and 9971 |
| Gastrointestinal complications | 9974 |
| Infection complications | 9985, 99859, and 99851 |
| Wound complications | 99883, 99812, 99813, 9986, and 9983 |
| Respiratory complications | 9973, 5185, 5121, 5185, and 5184 |
| Complications during the surgical procedure | 9982, 9984, and 99811 |
| Urinary complications | 9975 |
| Systemic complications | 9980 and 99889 |

Supplementary Table 2. Full sample

| **Variables** | **Hazard Ratio**  **(95% Confidence Interval)** | ***P* value** |
| --- | --- | --- |
| Insurance |  |  |
| Private | Reference |  |
| Medicaid | 0.958 (0.754-1.216) | 0.723 |
| Uninsured | 1.600 (1.239-2.067) | <0.001 |
| Age |  |  |
| 18-44 | Reference |  |
| 45-65 | 2.298 (1.606-3.289) | <0.001 |
| Sex |  |  |
| Male | Reference |  |
| Female | 0.831 (0.696-0.992) | 0.040 |
| Race |  |  |
| White | Reference |  |
| Black | 1.067 (0.829-1.375) | 0.614 |
| Hispanic | 0.844 (0.610-1.168) | 0.305 |
| Asian | 0.970 (0.587-1.603) | 0.905 |
| Native | 0.408 (0.059-2.829) | 0.364 |
| Other | 0.692 (0.368-1.303) | 0.254 |
| Income |  |  |
| Quart 1 | Reference |  |
| Quart 2 | 0.941 (0.744-1.191) | 0.614 |
| Quart 3 | 0.852 (0.664-1.093) | 0.207 |
| Quart 4 | 0.900 (0.686-1.181) | 0.447 |
| Hospital region |  |  |
| Northeast | Reference |  |
| Midwest | 1.195 (0.865-1.650) | 0.280 |
| South | 1.335 (1.037-1.717) | 0.024 |
| West | 1.336 (0.994-1.795) | 0.054 |
| Hospital size |  |  |
| Small | Reference |  |
| Medium | 0.952 (0.687-1.319) | 0.767 |
| Large | 0.888 (0.660-1.194) | 0.430 |
| Teaching status |  |  |
| Rural | Reference |  |
| Urban non-teaching | 0.592 (0.438-0.800) | <0.001 |
| Urban teaching | 0.477 (0.350-0.649) | <0.001 |
| Stage |  |  |
| Not node positive/metastatic | Reference |  |
| Node positive/metastatic | 1.528 (1.278-1.827) | <0.001 |
| Surgery type |  |  |
| Open | Reference |  |
| Laparoscopic | 0.399 (0.246-0.650) | <0.001 |
| Complications |  |  |
| Cardiovascular | 2.895 (2.116-3.959) | <0.001 |
| Gastrointestinal | 0.569 (0.428-0.757) | <0.001 |
| Infectious | 0.615 (0.461-0.821) | 0.001 |
| Wound | 0.497 (0.290-0.851) | 0.010 |
| Respiratory | 1.515 (1.103-2.080) | 0.010 |
| Surgical | 1.089 (0.758-1.564) | 0.644 |
| Urinary | 1.111 (0.605-2.042) | 0.734 |
| Systemic | 2.454 (1.523-3.954) | <0.001 |
| Year |  |  |
| 2005 | Reference |  |
| 2006 | 0.995 (0.658-1.504) | 0.981 |
| 2007 | 1.099 (0.740-1.632) | 0.639 |
| 2008 | 1.235 (0.854-1.787) | 0.261 |
| 2009 | 1.064 (0.711-1.593) | 0.762 |
| 2010 | 1.401 (0.957-2.051) | 0.083 |
| 2011 | 1.397 (0.941-2.073) | 0.097 |
| 2012 | 0.987 (0.652-1.494) | 0.949 |
| 2013 | 0.947 (0.623-1.441) | 0.800 |
| 2014 | 0.995 (0.660-1.498) | 0.979 |

Supplementary Table 3. Low Comorbidity

| **Variables** | **Hazard Ratio**  **(95% Confidence Interval)** | ***P* value** |
| --- | --- | --- |
| Insurance |  |  |
| Private | Reference |  |
| Medicaid | 1.384 (0.910-2.106) | 0.129 |
| Uninsured | 2.115 (1.421-3.149) | <0.001 |
| Age |  |  |
| 18-44 | Reference |  |
| 45-65 | 1.669 (0.985-2.829) | 0.056 |
| Sex |  |  |
| Male | Reference |  |
| Female | 1.064 (0.799-1.416) | 0.671 |
| Race |  |  |
| White | Reference |  |
| Black | 1.030 (0.646-1.641) | 0.902 |
| Hispanic | 0.442 (0.218-0.900) | 0.024 |
| Asian | 0.417 (0.148-1.174) | 0.097 |
| Native | 1.101 (0.157-7.717) | 0.923 |
| Other | 0.677 (0.251-1.831) | 0.442 |
| Income |  |  |
| Quart 1 | Reference |  |
| Quart 2 | 1.031 (0.680-1.564) | 0.884 |
| Quart 3 | 0.645 (0.404-1.032) | 0.067 |
| Quart 4 | 0.812 (0.519-1.269) | 0.360 |
| Hospital region |  |  |
| Northeast | Reference |  |
| Midwest | 0.736 (0.396-1.367) | 0.331 |
| South | 1.130 (0.746-1.711) | 0.564 |
| West | 1.553 (0.960-2.511) | 0.072 |
| Hospital size |  |  |
| Small | Reference |  |
| Medium | 0.752 (0.449-1.260) | 0.279 |
| Large | 0.572 (0.357-0.917) | 0.020 |
| Teaching status |  |  |
| Rural | Reference |  |
| Urban non-teaching | 0.585 (0.349-0.980) | 0.041 |
| Urban teaching | 0.468 (0.273-0.802) | 0.005 |
| Stage |  |  |
| Not node positive/metastatic | Reference |  |
| Node positive/metastatic | 1.478 (1.093-1.999) | 0.011 |
| Surgery type |  |  |
| Open | Reference |  |
| Laparoscopic | 0.198 (0.062-0.634) | 0.006 |
| Complications |  |  |
| Cardiovascular | 3.274 (1.811-5.918) | <0.001 |
| Gastrointestinal | 0.607 (0.380-0.969) | 0.036 |
| Infectious | 0.759 (0.472-1.222) | 0.256 |
| Wound | 0.646 (0.247-1.687) | 0.371 |
| Respiratory | 2.007 (1.131-3.561) | 0.017 |
| Surgical | 1.150 (0.651-2.033) | 0.629 |
| Urinary | 1.679 (0.690-4.083) | 0.253 |
| Systemic | 3.340 (1.498-7.448) | 0.003 |
| Year |  |  |
| 2005 | Reference |  |
| 2006 | 0.581 (0.313-1.076) | 0.084 |
| 2007 | 1.190 (0.708-2.001) | 0.510 |
| 2008 | 1.059 (0.615-1.825) | 0.835 |
| 2009 | 0.622 (0.307-1.258) | 0.186 |
| 2010 | 1.383 (0.772-2.478) | 0.275 |
| 2011 | 1.059 (0.522-2.148) | 0.874 |
| 2012 | 0.676 (0.327-1.398) | 0.290 |
| 2013 | 0.608 (0.283-1.307) | 0.202 |
| 2014 | 0.425 (0.179-1.010) | 0.052 |

Supplementary Table 4. Low comorbidity in urban teaching hospitals

| **Variables** | **Hazard Ratio**  **(95% Confidence Interval)** | ***P* value** |
| --- | --- | --- |
| Insurance |  |  |
| Private | Reference |  |
| Medicaid | 1.656 (0.908-3.021) | 0.100 |
| Uninsured | 2.278 (1.246-4.161) | 0.007 |
| Age |  |  |
| 18-44 | Reference |  |
| 45-65 | 2.283 (1.024-5.089) | 0.043 |
| Sex |  |  |
| Male | Reference |  |
| Female | 1.041 (0.702-1.542) | 0.843 |
| Race |  |  |
| White | Reference |  |
| Black | 0.874 (0.476-1.605) | 0.664 |
| Hispanic | 0.159 (0.038-0.667) | 0.012 |
| Asian | 0.389 (0.117-1.298) | 0.124 |
| Native | 2.231 (0.347-14.321) | 0.397 |
| Other | 0.268 (0.036-1.973) | 0.195 |
| Income |  |  |
| Quart 1 | Reference |  |
| Quart 2 | 1.546 (0.793-3.016) | 0.201 |
| Quart 3 | 0.878 (0.429-1.797) | 0.721 |
| Quart 4 | 1.153 (0.595-2.236) | 0.673 |
| Hospital region |  |  |
| Northeast | Reference |  |
| Midwest | 0.908 (0.354-2.329) | 0.841 |
| South | 1.455 (0.815-2.596) | 0.204 |
| West | 2.289 (1.197-4.378) | 0.012 |
| Hospital size |  |  |
| Small | Reference |  |
| Medium | 0.675 (0.342-1.334) | 0.258 |
| Large | 0.487 (0.252-0.942) | 0.032 |
| Stage |  |  |
| Not node positive/metastatic | Reference |  |
| Node positive/metastatic | 1.319 (0.845-2.059) | 0.223 |
| Surgery type |  |  |
| Open | Reference |  |
| Laparoscopic | 0.477 (0.145-1.564) | 0.221 |
| Complications |  |  |
| Cardiovascular | 2.793 (1.304-5.980) | 0.008 |
| Gastrointestinal | 0.566 (0.285-1.121) | 0.102 |
| Infectious | 0.839 (0.465-1.515) | 0.561 |
| Wound | 0.611 (0.168-2.216) | 0.453 |
| Respiratory | 1.845 (0.805-4.225) | 0.147 |
| Surgical | 0.787 (0.323-1.916) | 0.597 |
| Urinary | 2.611 (0.881-7.737) | 0.083 |
| Systemic | 3.101 (0.873-11.012) | 0.080 |
| Year |  |  |
| 2005 | Reference |  |
| 2006 | 0.783 (0.333-1.838) | 0.574 |
| 2007 | 1.093 (0.506-2.360) | 0.820 |
| 2008 | 1.028 (0.457-2.312) | 0.945 |
| 2009 | 0.533 (0.196-1.444) | 0.215 |
| 2010 | 0.985 (0.397-2.443) | 0.974 |
| 2011 | 0.720 (0.275-1.889) | 0.504 |
| 2012 | 0.261 (0.070-0.968) | 0.044 |
| 2013 | 0.300 (0.089-1.011) | 0.052 |
| 2014 | 0.423 (0.149-1.204) | 0.106 |
